# Relationship between the Chinese visceral adiposity index and gout in individuals with type 2 diabetes mellitus: a cross-sectional population-based study

**DOI:** 10.3389/fnut.2025.1697822

**Published:** 2025-11-21

**Authors:** Ningyu Cai, Mengdie Chen, Ping Feng, Yiyun Wang, Xianping Zhu, Qidong Zheng

**Affiliations:** 1 Department of Orthopedics, Taizhou Central Hospital (Taizhou University Hospital), Zhejiang, China; 2 Department of Endocrinology, Taizhou Central Hospital (Taizhou University Hospital), Zhejiang, China; 3 Department of Internal Medicine, Yuhuan Second People’s Hospital, Zhejiang, China

**Keywords:** Chinese Visceral Adiposity Index, gout, type 2 diabetes mellitus, risk factors, obesity

## Abstract

**Background:**

Visceral adiposity indices have recently attracted considerable attention in metabolic and cardiovascular research. Here, the link between the Chinese Visceral Adiposity Index (CVAI) and gout in individuals with type 2 diabetes mellitus (T2DM) was investigated.

**Methods:**

This cross-sectional analysis evaluated 14,099 adults with T2DM. The CVAI-gout relationship was investigated using restricted cubic spline (RCS) modeling to assess dose-responsiveness. Multivariable logistic regression was applied, with adjustments for demographic and metabolic covariates. Subgroup analyses and interaction tests were performed across clinically relevant strata, and ROC curves were applied to assess the utility of the CVAI in discriminate patients with gout.

**Results:**

Among all participants, 5.7% were diagnosed with gout. Higher CVAI values were linked with raised gout incidence (adjusted OR 1.07, 95% CI 1.05–1.09), with RCS models revealing a steadily increasing, positive association between between the CVAI and gout prevalence. After full adjustment, the top CVAI quartile showed a 2.44-fold greater prevalence of gout (95% CI 1.85–3.22) relative to the lowest quartile. Subgroup analyses confirmed consistent associations across various categories. ROC analysis further demonstrated that CVAI had superior discriminative ability for gout compared with traditional obesity indices.

**Conclusion:**

This large-sample investigation of Chinese patients with T2DM indicated a strong positive link between the CVAI and gout, suggesting the potential of the CVAI for stratifying metabolic risk in this population.

## 1 Introduction

Diabetes mellitus (DM) poses a significant and escalating threat to public health worldwide, with China currently bearing the heaviest national burden. More than 118 million Chinese adults are affected by diabetes, representing approximately 22% of all cases worldwide (1). Alongside the rising prevalence of diabetes, gout, a metabolic and inflammatory arthritic disorder resulting from hyperuricemia and the deposition of monosodium urate crystals, has also increased steadily, mirroring global trends in obesity (2). Patients with type 2 diabetes mellitus (T2DM) are particularly susceptible, facing an estimated 48% higher likelihood of developing gout compared with age- and sex-matched counterparts without T2DM (3). The coexistence of diabetes and gout significantly amplifies the risk of cardiovascular complications (4), renal dysfunction (5), and limb amputation (6), while further aggravating metabolic imbalances. These combined effects pose substantial challenges for clinical management (7). Therefore, early detection of diabetic individuals at increased risk of gout is of critical clinical importance to enable timely and personalized therapeutic strategies.

Gout is a multifactorial metabolic disorder, with obesity recognized as one of its principal risk factors (8). Among different fat depots, visceral adipose tissue (VAT) is especially influential in determining gout susceptibility (9). Functioning as an active endocrine organ, VAT secretes inflammatory adipokines and free fatty acids that promote insulin resistance, systemic inflammation, and dyslipidemia, all of which promote hyperuricemia and gout development (10, 11). Although imaging modalities such as magnetic resonance imaging (MRI) and ultrasonography can directly quantify VAT, their high cost, technical complexity, and limited accessibility hinder widespread clinical application (12). To overcome these limitations, several surrogate indices have been developed. Conventional measures such as body mass index (BMI) and waist circumference (WC) are convenient but fail to accurately reflect visceral fat distribution (13).

To improve the assessment of adipose tissue dysfunction, the Visceral Adiposity Index (VAI) was introduced, combining BMI, WC, triglycerides (TG), and high-density lipoprotein cholesterol (HDL-C) levels into a composite score (14). However, assessments of potential associations between the VAI and hyperuricemia have yielded inconsistent findings (15, 16): some report positive associations in US populations (15), while others in Chinese cohorts report inverse or non-significant relationships (16). Recognizing ethnic and metabolic differences, researchers subsequently developed the Chinese Visceral Adiposity Index (CVAI). This population-specific, sex-adjusted index incorporates WC, BMI, TG, and HDL-C to more accurately reflect visceral fat accumulation in Chinese adults (17). Previous research has established independent links between raised CVAI and elevated risks of cardiovascular/cerebrovascular disease and hypertension (18–20). Despite these promising observations, there is limited evidence on the relationship between the CVAI and gout.

Here, a dual-center cross-sectional investigation of 14,099 patients with T2DM was conducted to evaluate the links between the CVAI and gout prevalence. The findings are expected to improve risk stratification and inform early prevention and treatment.

## 2 Materials and methods

### 2.1 Study description

**FIGURE 1:**
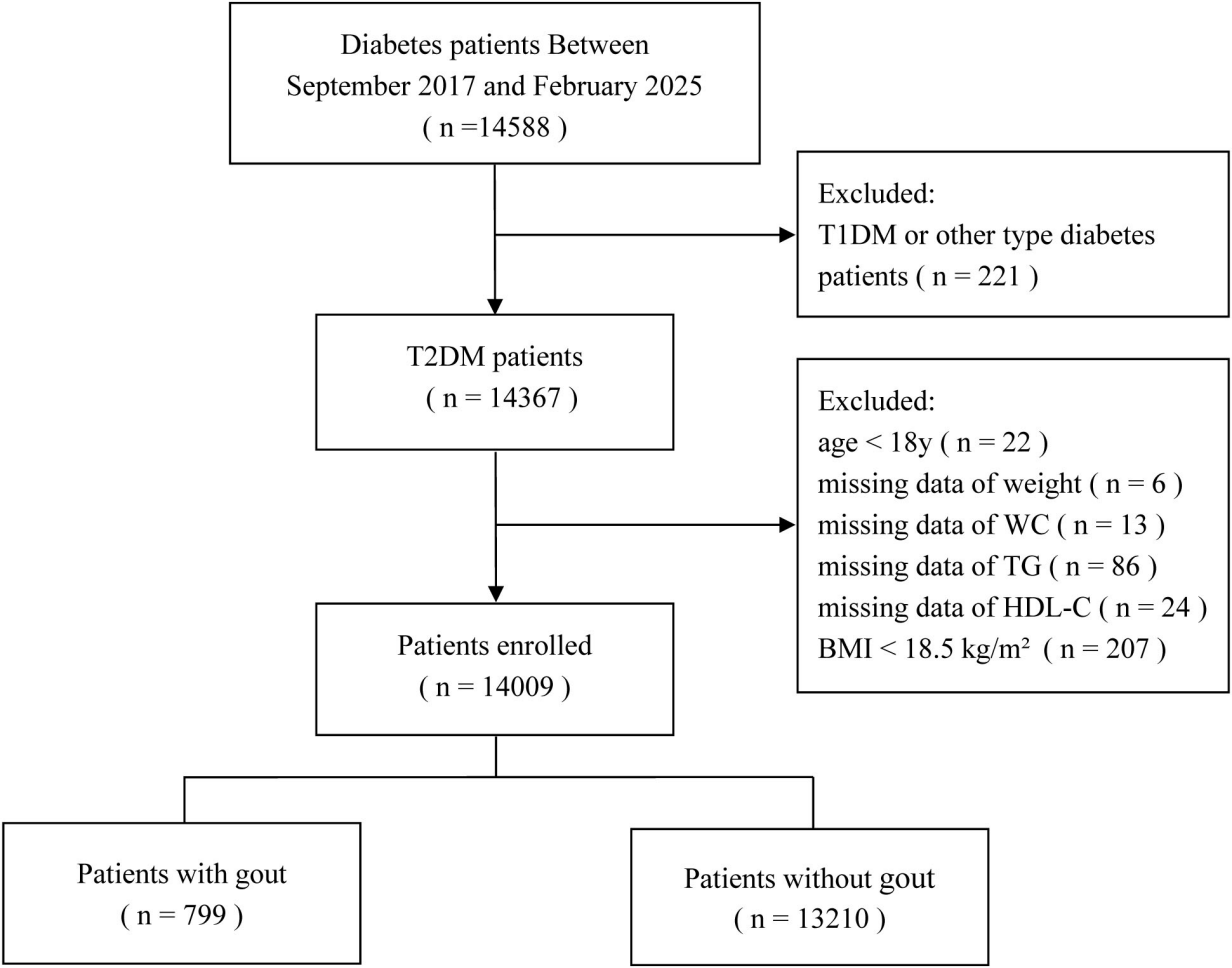
Flow diagram of participants selection. T1DM, type 1 diabetes mellitus; T2DM, type 2 diabetes mellitus; WC, waist circumference; TG, triglycerides; HDL-C, high-density lipoprotein cholesterol; BMI, body mass index.

This cross-sectional analysis evaluated data from the National Metabolic Management Center (MMC) at Taizhou Central Hospital (Taizhou University Hospital) and Yuhuan Second People’s Hospital, using data from June 2017 to February 2025. Individuals were considered they were ≥ 18 years and had been clinically diagnosed with T2DM according to the WHO criteria (21). Those with type 1 diabetes, other diabetes types, or missing data on key variables, such as body weight, WC, TG, or HDL-C, or BMI < 18.5 kg/m^2^, were excluded. Ultimately, 14,099 participants were enrolled. The participants selection process is depicted in Figure 1. The protocol was approved by the institutional review boards of both hospitals, and written informed consent was provided by all participants before enrollment.

### 2.2 Data collection

Questionnaires were administered by trained personnel, collecting demographic information (age and sex), educational level, diabetes duration, smoking and alcohol consumption history, and physician-diagnosed comorbidities such as hypertension and dyslipidemia. All physical examinations were performed under standardized conditions. Weight, height, and WC were assessed using calibrated instruments, and BMI values were assessed as weight (kg) divided by the height squared (m^2^). Blood pressure was recorded twice following sitting for a minimum of 5 min, determining both the average systolic (SBP) and diastolic (DBP) values.

### 2.3 Laboratory measurements

Measurements were performed on fasting venous blood, assessing the levels of fasting plasma glucose (FPG), fasting serum C-peptide (FCp), glycated hemoglobin (HbA1c), alanine aminotransferase (ALT), aspartate aminotransferase (AST), serum uric acid (UA), serum creatinine (Scr), urinary albumin-to-creatinine ratio (UACR), total cholesterol (TC), TG, HDL-C, and low-density lipoprotein cholesterol (LDL-C). The estimated glomerular filtration rate (eGFR) was determined using the CKD-EPI equation (22).

### 2.4 Definition

The formulae used for VAI calculation were as follows (14):

Males: WC (cm)/(39.68 + 1.88 × BMI (kg/m^2^)) × TG/1.03 × 1.31/HDL-C (mmol/L),
Females: WC (cm)/(36.58 + 1.89 × BMI (kg/m^2^)) × TG/0.81 × 1.52/HDL-C (mmol/L).

The formulae used for CVAI calculation were as follows (17):

Males: −267.93 + 0.68 × age (years) + 0.03 × BMI (kg/m^2^) + 4.00 × WC (cm) + 22.00 × log10 TG (mmol/L) − 16.32 × HDL-C (mmol/L),
Females: − 187.32 + 1.71 × age (years) + 4.23 × BMI (kg/m^2^) + 1.12 × WC (cm) + 39.76 × log10 TG (mmol/L) − 11.66 × HDL-C (mmol/L).

CVAI values were classified into four quartiles.

Gout was diagnosed according to the 1977 American College of Rheumatology (ACR) classification criteria (23).

### 2.5 Statistical analyses

Data were analyzed using R (version 4.2.2) and Free Statistics software (version 2.2.0), with two-sided *P* < 0.05 representing statistical significance. Categorical variables are shown as counts and percentages, while continuous data are given as medians with interquartile ranges (IQRs). Differences among the CVAI quartiles were examined using Kruskal-Wallis and chi-square tests for continuous and categorical data, respectively.

The CVAI-gout relationship was assessed using multivariable logistic regression. Four models were constructed: (1) a model with no adjustments; (2) Model 1, with adjustments for age and sex; (3) Model 2, with additional adjustments for education, smoking, alcohol consumption; and (4) Model 3, further adjusted for SBP, FCp, HbA1c, UA, and eGFR. CVAI was analyzed both as a continuous variable (with odds ratio [OR] calculated per 10 units increase) and as categorical quartiles, with the bottom quartile, Q1, representing the reference. For analysis of trends, quartile medians were utilized as continuous variables. Results are expressed as ORs with 95% CIs.

To assess the linearity of the relationships, restricted cubic spline (RCS) regression models were fitted with knots at the 5th, 35th, 65th, and 95th percentiles of the CVAI distribution. Subgroup analyses were undertaken using stratified logistic regression, with adjustment for the covariates included in Models 1 and 3. Possible effect modifications were examined by introducing multiplicative interaction terms between CVAI and subgroup variables, specifically, sex, age (< 65 vs. ≥ 65 years), educational level (less than high school vs. high school or higher), HbA1c (< 7% vs. ≥ 7%), BMI (< 24 vs. 24–28 vs. ≥ 28kg/m^2^), smoking status, and alcohol consumption. Bonferroni correction was used to correct for multiple testing. The discriminative ability of CVAI, VAI, BMI and WC for identifying gout among patients with T2DM was further evaluated using receiver operating characteristic (ROC) curve analysis, with the area under the curve (AUC) and corresponding 95% CIs used to quantify the discriminative ability of each index. The DeLong test was utilized to compare the AUC values of the CVAI with those of three other related indices, aiming to ascertain whether the CVAI exhibits superior predictive performance. Additionally, the Net Reclassification Index (NRI) and the Integrated Discrimination Improvement (IDI) index were employed to further evaluate the incremental predictive value of the CVAI, VAI, BMI and WC relative to the basic models.

## 3 Results

### 3.1 Participant characteristics

Overall, 14,099 participants with T2DM were enrolled, among whom 799 (5.7%) were diagnosed with gout. Table 1 details their baseline information and quartile divisions. Those in the upper CVAI quartiles were more often older and male. Measures of adiposity, including BMI, WC, and VAI, increased progressively with higher CVAI levels (all *P* < 0.001). Across ascending CVAI quartiles, participants also demonstrated a gradual decline in renal function, a higher likelihood of albuminuria, and a raised frequency of comorbid hypertension and dyslipidemia. Serum uric acid concentrations rose significantly from the lowest to the highest quartile, accompanied by a significant rise in gout prevalence (Q1: 2.2% vs. Q4: 10%, *P* < 0.001).

**TABLE 1:** Baseline participant information stratified by CVAI quartiles.

Variables	CVAI					*P*-value
	Total (*n* = 14,009)	Q1 (*n* = 3,502)	Q2 (*n* = 3,502)	Q3 (*n* = 3,502)	Q4 (*n* = 3,503)	
Male, n (%)	8,474 (60.5)	1,867 (53.3)	1,965 (56.1)	2,115 (60.4)	2,527 (72.1)	< 0.001
Age, y	55.0 (47.0, 64.0)	52.0 (44.0, 58.0)	55.0 (48.0, 62.0)	58.0 (50.0, 66.0)	58.0 (48.0, 68.0)	< 0.001
High school and above, n (%)	2,294 (16.4)	627 (17.9)	539 (15.4)	530 (15.1)	598 (17.1)	0.004
DBP (mmHg)	75.0 (69.0, 83.0)	74.0 (68.0, 80.0)	75.0 (69.0, 82.0)	76.0 (69.0, 84.0)	78.0 (71.0, 86.0)	< 0.001
SBP (mmHg)	130.0 (120.0, 141.0)	124.0 (115.0, 135.0)	128.0 (120.0, 140.0)	131.0 (122.0, 143.0)	135.0 (126.0, 146.0)	< 0.001
Weight (kg)	67.8 (60.0, 76.0)	59.0 (54.1, 64.6)	65.4 (59.5, 71.0)	70.4 (64.0, 76.4)	79.4 (71.8, 88.1)	< 0.001
BMI (kg/m^2^)	25.2 (23.1, 27.6)	22.4 (21.0, 23.9)	24.4 (23.1, 25.8)	26.0 (24.6, 27.5)	28.7 (26.8, 31.0)	< 0.001
WC (cm)	89.7 (83.6, 96.0)	80.0 (76.0, 83.0)	87.0 (84.8, 89.5)	92.0 (90.0, 95.0)	100.0 (97.0, 104.5)	< 0.001
VAI	2.2 (1.4, 3.8)	1.4 (0.9, 2.2)	2.1 (1.4, 3.3)	2.7 (1.8, 4.2)	3.3 (2.1, 5.5)	< 0.001
Duration of diabetes (y)	3.2 (0.2, 10.1)	3.0 (0.2, 9.3)	3.4 (0.2, 10.2)	3.5 (0.2, 10.2)	3.2 (0.1, 10.2)	0.003
Hypertension, n (%)	7,819 (55.8)	1,202 (34.3)	1,870 (53.4)	2,195 (62.7)	2,552 (72.9)	< 0.001
Dyslipidemia, n (%)	9,499 (67.8)	1,793 (51.2)	2,324 (66.4)	2,568 (73.3)	2,814 (80.3)	< 0.001
Gout, n (%)	799 (5.7)	77 (2.2)	135 (3.9)	237 (6.8)	350 (10)	< 0.001
Smoking, n (%)	3,472 (24.8)	756 (21.6)	787 (22.5)	862 (24.6)	1,067 (30.5)	< 0.001
Alcohol consumption, n (%)	1,909 (13.6)	374 (10.7)	438 (12.5)	491 (14)	606 (17.3)	< 0.001
FBG (mmol/L)	8.2 (6.6, 11.0)	8.2 (6.5, 11.5)	8.1 (6.6, 10.9)	8.1 (6.6, 10.7)	8.4 (6.7, 11.1)	0.02
FCp (ng/mL)	2.1 (1.5, 2.9)	1.6 (1.1, 2.1)	2.0 (1.5, 2.6)	2.3 (1.7, 3.0)	2.8 (2.0, 3.7)	< 0.001
HbA1c (%)	8.2 (6.9, 10.3)	8.4 (6.8, 10.9)	8.2 (6.9, 10.3)	8.2 (6.9, 9.9)	8.3 (7.0, 10.0)	0.013
ALT (U/L)	23.0 (16.0, 36.0)	19.0 (14.0, 28.0)	22.0 (16.0, 34.0)	24.0 (17.0, 38.0)	28.0 (19.0, 47.0)	< 0.001
AST (U/L)	20.0 (16.0, 26.0)	18.0 (15.0, 23.0)	19.0 (16.0, 25.0)	20.0 (16.0, 27.0)	22.0 (17.0, 31.0)	< 0.001
BUN (mmol/L)	5.3 (4.4, 6.5)	5.2 (4.2, 6.2)	5.3 (4.3, 6.4)	5.4 (4.4, 6.6)	5.5 (4.5, 6.7)	< 0.001
Scr (μmol/L)	63.0 (52.0, 76.0)	58.0 (48.0, 71.0)	61.0 (51.0, 73.0)	65.0 (54.0, 77.0)	68.0 (57.0, 81.0)	< 0.001
eGFR (mL/min per 1.73 m^2^)	104.5 (85.2, 125.5)	110.7 (92.8, 133.5)	106.3 (88.3, 126.4)	101.1 (81.8, 121.3)	99.5 (78.2, 120.7)	< 0.001
UA (μmol/L)	328.0 (268.0, 397.0)	293.0 (242.0, 353.0)	317.0 (263.0, 379.8)	338.0 (279.0, 407.0)	367.0 (304.0, 438.0)	< 0.001
TG (mmol/L)	1.6 (1.1, 2.4)	1.1 (0.8, 1.6)	1.5 (1.1, 2.2)	1.7 (1.2, 2.6)	2.1 (1.4, 3.2)	< 0.001
TC (mmol/L)	5.0 (4.2, 5.9)	5.0 (4.2, 5.8)	5.1 (4.3, 5.9)	5.0 (4.2, 5.9)	5.1 (4.2, 6.0)	0.002
HDLc (mmol/L)	1.1 (0.9, 1.3)	1.2 (1.0, 1.5)	1.1 (0.9, 1.3)	1.0 (0.9, 1.2)	1.0 (0.8, 1.1)	< 0.001
LDLc (mmol/L)	2.9 (2.2, 3.6)	2.9 (2.3, 3.6)	3.0 (2.3, 3.6)	2.9 (2.2, 3.5)	2.8 (2.1, 3.5)	< 0.001
UACR (mg/g)	20.0 (8.7, 58.5)	15.8 (7.5, 41.2)	18.6 (8.5, 54.0)	20.5 (8.9, 60.5)	27.1 (11.1, 83.4)	< 0.001

CVAI, Chinese visceral adiposity index; SBP, systolic blood pressure; DBP, diastolic blood pressure; BMI, body mass index; WC, waist circumference; VAI, visceral adiposity index; FBG, fasting blood glucose; FCp, fasting serum C peptide; HbA1c, glycated hemoglobin; ALT, alanine transaminase; AST, aspartate aminotransferase; BUN, urea nitrogen; Scr, serum creatinine; eGFR, estimated glomerular filtration rate; UA, uric acid; TG, triglycerides; TC, total cholesterol; HDL-C, high-density lipoprotein cholesterol; LDL-C, low-density lipoprotein cholesterol; UACR, urinary albumin-to-creatinine ratio.

### 3.2 Relationship between CVAI and gout in T2DM cases

The logistic regression results are summarized in Table 2. In the absence of adjustment, each 10 units increases in CVAI showed marked associations with greater odds of gout (OR = 1.13, 95% CI: 1.11–1.15, *P* < 0.001). Analysis of CVAI as a categorical variable showed that cases in the top quartile, Q4, had a 4.85-fold increased likelihood of gout relative to those in the bottom quartile, Q1 (OR = 4.85, 95% CI: 3.75–6.28, *P* < 0.001). Following adjustments for age and sex (Model 1), and further adjustment for education level, smoking, alcohol consumption (Model 2), this relationship remained robust. Further adjustment for additional metabolic and renal factors in Model 3 slightly attenuated the effect size but maintained statistical significance (per 10 units OR = 1.07, 95% CI: 1.05–1.09, *P* < 0.001; Q4 vs. Q1: OR = 2.44, 95% CI: 1.85–3.22, *P* < 0.001).

**TABLE 2:** Association between CVAI and gout among participants with T2DM.

Model variable	Crude		Model 1		Model 2		Model 3	
	OR (95% CI)	*P*-value	OR (95% CI)	*P*-value	OR (95% CI)	*P*-value	OR (95% CI)	*P*-value
CVAI	1.13 (1.11∼1.15)	< 0.001	1.12 (1.1∼1.14)	< 0.001	1.11 (1.09∼1.13)	< 0.001	1.07 (1.05∼1.09)	< 0.001
**Subgroups**								
Q1	1(Ref)		1(Ref)		1(Ref)			
Q2	2.2 (1.66∼2.92)	< 0.001	1.98 (1.49∼2.64)	< 0.001	1.97 (1.48∼2.61)	< 0.001	1.65 (1.23∼2.21)	0.001
Q3	3.47 (2.66∼4.53)	< 0.001	3.07 (2.35∼4.02)	< 0.001	3.02 (2.31∼3.96)	< 0.001	2.17 (1.64∼2.87)	< 0.001
Q4	4.85 (3.75∼6.28)	<0.001	4.13 (3.18∼5.37)	<0.001	4.05 (3.12∼5.27)	<0.001	2.44 (1.85∼3.22)	<0.001
P for trend		< 0.001		<0.001		< 0.001		<0.001

CVAI, Chinese visceral adiposity index; T2DM, type 2 diabetes mellitus; OR, odd ratio; CI, confidence interval. CVAI was entered as a continuous variable per 10 units increase. Crude: no adjustments; Model 1: adjustments for sex, age; Model 2: further adjustments for education level, smoking, alcohol consumption; Model 3: further adjustments for SBP, FCp, HbA1c, UA, and eGFR.

**FIGURE 2:**
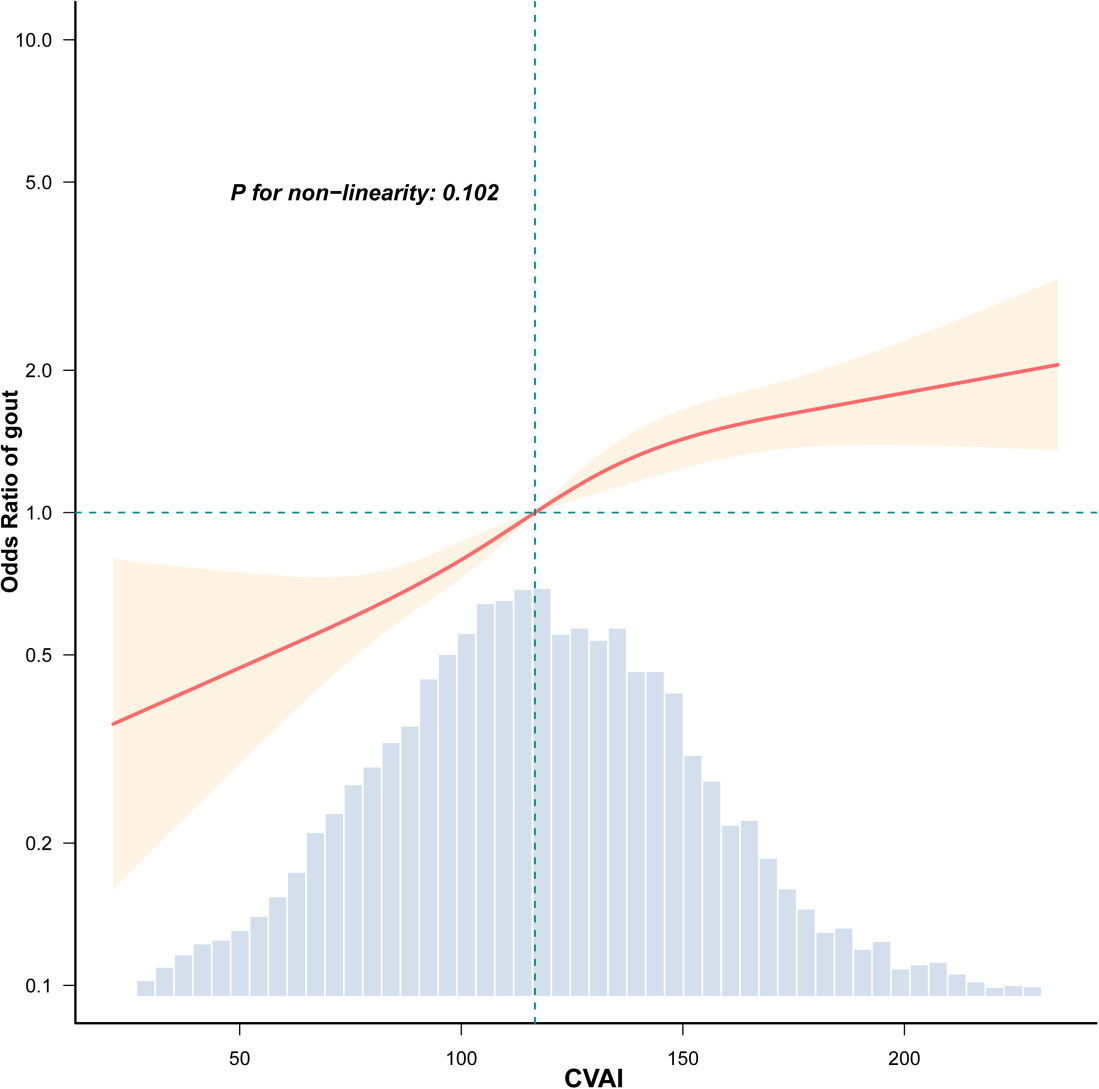
Relationship between CVAI and gout prevalence among individuals with T2DM. RCS regression results (knots: 5th, 35th, 65th, and 95th percentiles). The shaded area indicates 95% CIs. Adjustments were made for age, sex, education level, smoking, alcohol consumption, SBP, FCp, HbA1c, UA, and eGFR. Only the central 99% of the data distribution is shown.

Trend analyses consistently demonstrated a marked dose-dependent link between rising CVAI levels and gout prevalence across all models (*P* for trend < 0.001). RCS analyses further confirmed that the CVAI-gout prevalence association was approximately linear (Figure 2; *P* for non-linearity = 0.102).

### 3.3 Subgroup analysis

**FIGURE 3:**
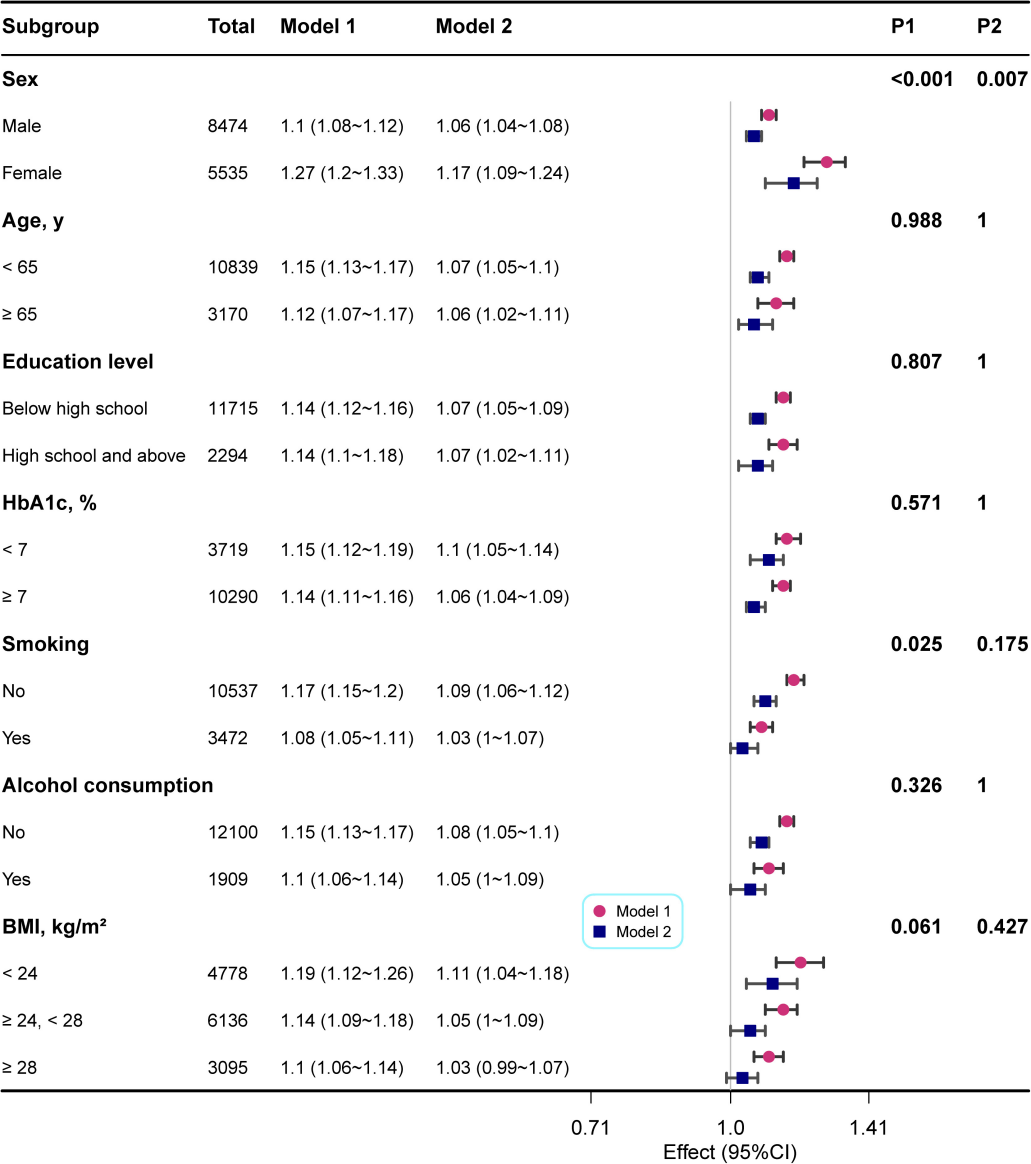
Forest plots showing results of the subgroup analysis of the CVAI-gout link in individuals with T2DM. ORs and 95% CIs are shown for the different subgroups. Model 1: unadjusted; Model 2: adjusted for age, sex, educational level, smoking, alcohol consumption, SBP, FCp, HbA1c, UA, and eGFR; P1: unadjusted P for interaction; P2: adjusted P for interaction (Bonferroni).

As illustrated in Figure 3, stratified analyses demonstrated a consistent positive relationship between CVAI and gout prevalence over all subgroups. In Model 1, the associations remained uniformly significant, whereas in the fully adjusted Model 2, the effect sizes were slightly attenuated but remained statistically significant. The stratified analysis revealed significant interaction effects for sex, while no significant effect modification was observed for age, educational level, HbA1c, BMI, or alcohol consumption (P for interaction > 0.05). Although a nominal interaction with smoking was noted (*P* = 0.025), it did not retain statistical significance after adjustment (Bonferroni correction) for multiple comparisons (*P* = 0.175). Furthermore, restricted cubic spline analyses confirmed a linear positive dose-response relationship between CVAI and gout prevalence in both males and females (Supplementary Figure 1).

### 3.4 ROC analysis

**FIGURE 4:**
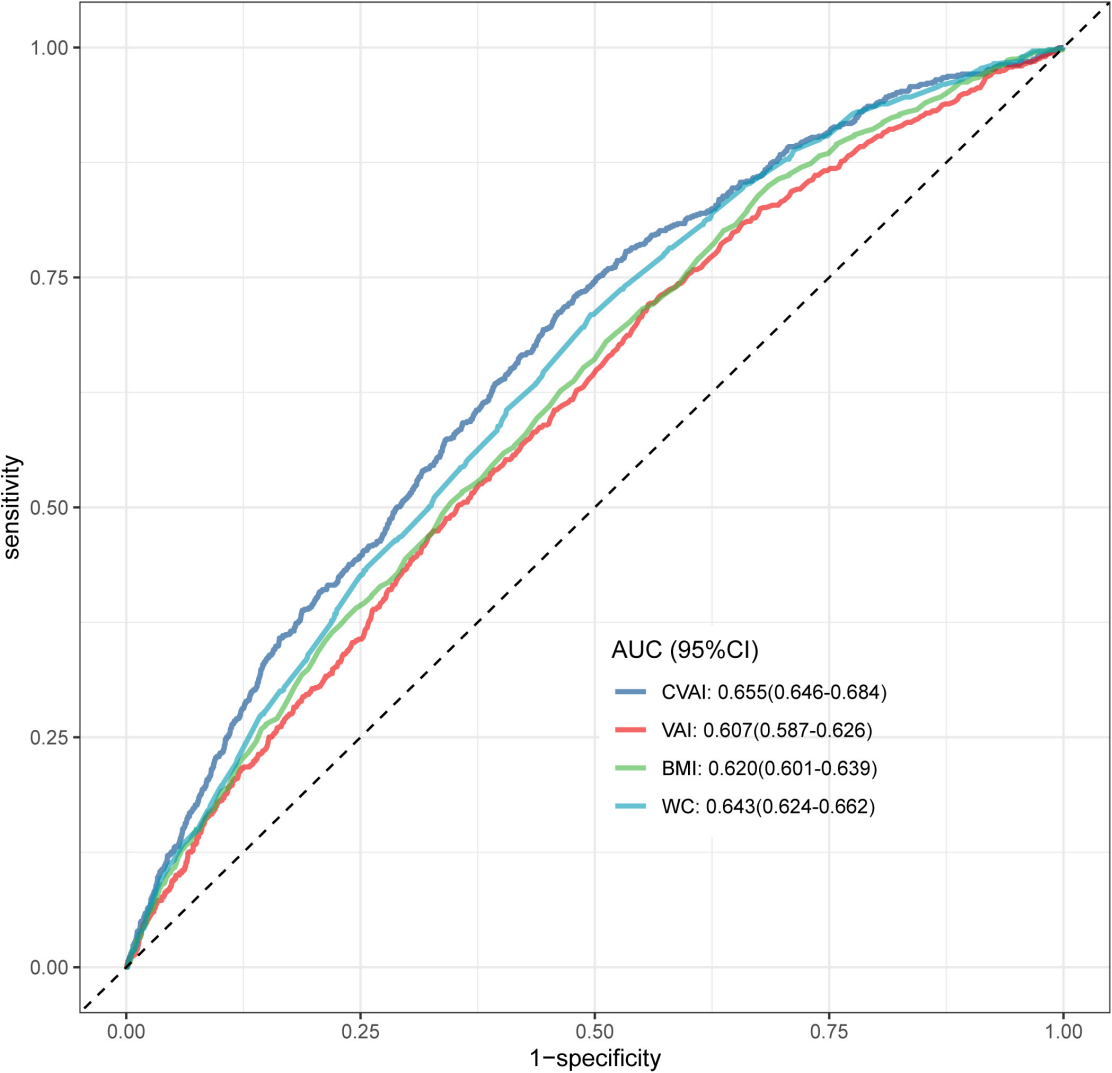
ROC curves of the CVAI, VAI, BMI, and WC in identifying gout in T2DM participants. ROC, receiver operating characteristic; VAI, visceral adiposity index; CVAI, Chinese visceral adiposity index; BMI, body mass index; WC, waist circumference.

The discriminative ability of CVAI and conventional obesity indices for identifying gout was evaluated using ROC curve analysis (Figure 4). Among all indices assessed, CVAI demonstrated the highest AUC of 0.655(95% CI 0.646–0.684), surpassing BMI, VAI, and WC (all *P* < 0.001) in distinguishing individuals with T2DM who had gout (Table 3). Consistent with these findings, Supplementary Table 1 quantifies the incremental utility using IDI and NRI values. CVAI significantly improved risk reclassification, NRI = 0.248 (95% CI 0.177–0.319) and IDI = 0.004 (95% CI 0.002–0.006) (both *P* < 0.001), outperforming all other related indices.

**TABLE 3:** ROC analysis comparing the ability of the CVAI and traditional obesity indices to identifying gout in T2DM participants.

Test	AUC (95%CI)	Best threshold	Specificity	Sensitivity	*P*-value
CVAI	0.655(0.646–0.684)	118.895	0.539	0.712	–
VAI	0.607(0.587–0.626)	1.995	0.443	0.721	< 0.001
BMI	0.620(0.601–0.639)	25.08	0.488	0.681	< 0.001
WC	0.643(0.624–0.662)	89.15	0.504	0.710	<0.001

ROC, receiver operating characteristic; T2DM, type 2 diabetes mellitus; AUC, area under the curve; CVAI, Chinese visceral adiposity index; VAI, visceral adiposity index; BMI, body mass index; WC, waist circumference. Delong’s test *p*-value comparing each index with CVAI.

## 4 Discussion

This large cross-sectional evaluation of 14,099 Chinese adults with T2DM demonstrated a strong and consistent relationship between higher CVAI values and increased likelihood of gout. This relationship persisted even after comprehensive adjustment for demographic, metabolic, and renal confounders and remained robust across all examined subgroups. CVAI demonstrated superior discriminative ability relative to conventional indices of obesity in identifying gout among patients with T2DM.

Our findings align with and extend previous evidence linking visceral adiposity to hyperuricemia and gout, as reported in studies employing both imaging modalities and surrogate adiposity markers. For example, a case–control study found that visceral fat obesity (VFO) independently predicted primary gout among metabolically obese but normal-weight individuals (adjusted OR = 2.488, 95% CI: 1.041–4.435) (24). Similarly, in men without metabolic syndrome, VAI was identified as an independent predictor of hyperuricemia in a cross-sectional analysis (25). Supporting these observations, Dong et al. (26) utilized information from the 2009 China Health and Nutrition Survey, demonstrating that VAI more accurately predicted hyperuricemia than traditional obesity indices, irrespective of general adiposity patterns. Consistent with these results, analyses of US NHANES data also revealed a dose-dependent increase in both hyperuricemia and gout prevalence across higher VAI quartiles (15).

The CVAI includes five factors, WC, BMI, age, TG, and HDL-C, each of which is biologically relevant to urate metabolism and the pathogenesis of gout. Our previous work demonstrated linear associations between both WC and BMI and gout prevalence in individuals with diabetes, yielding adjusted odds ratios of 1.775 (95% CI: 1.468–2.145) and 1.691 (95% CI: 1.394–2.053), respectively (8). These results align with several prospective analyses that have identified BMI as a key determinant of gout development (27, 28). Age also strongly influences gout burden. Data from NHANES revealed that gout prevalence rises sharply with advancing age, from 0.7% among adults aged 20–39 years to nearly 9% among those aged 80 years or older (29). Dyslipidemic indices play a significant role in gout risk. For instance, the TG-to-HDL-C ratio, a widely used surrogate indicator of insulin resistance, showed a J-shaped relationship with gout incidence in a nationwide cohort, regardless of diabetes status (30). Similarly, the ratio between non-HDL-C and HDL-C independently predicted gout, with each unit increase linked to a 10% rise in risk (adjusted OR = 1.10, 95% CI: 1.04–1.16) (31). Mechanistic investigations further support these epidemiologic findings, indicating that elevated VLDL triglycerides may intensify gout risk, particularly in individuals with hyperuricemia (32).

Although direct investigations of the relationship between CVAI and gout remain limited, there have several evaluations of the link between CVAI and hyperuricemia. Luo et al. (33), in an analysis of more than 22,000 adults from the CMEC cohort, reported consistent positive relationships between raised CVAI and hyperuricemia in males and females. Similarly, prospective findings from the CHARLS cohort indicated that one standard deviation increases in CVAI were linked to a 56% greater 4-year risk of developing hyperuricemia (adjusted HR = 1.56, 95% CI: 1.32–1.85), with CVAI outperforming conventionally used indices of adiposity (34).

In another large cross-sectional study comprising 10,016 participants, a J-shaped association between dose and response was found, with individuals in the top CVAI quartile demonstrating a 2.66-fold increased likelihood of hyperuricemia development relative to those in the bottom quartile (35). Comparable trends have been reported in diabetic populations. For instance, a cross-sectional investigation of 2,268 participants with T2DM demonstrated a progressive increase in the prevalence of hyperuricemia across CVAI quartiles, and ROC curve analysis confirmed the superior discriminative performance of CVAI relative to traditional adiposity measures (AUC = 0.83 vs. 0.71–0.78) (36). Wang et al. (37) found a marked dose-dependent link between higher CVAI and greater likelihood of hyperuricemia in 1,588 hospitalized T2DM patients.

The frequent co-occurrence of diabetes and gout can be attributed to several interconnected pathological mechanisms. Firstly, hyperglycemia competitively inhibits the excretion of uric acid in the renal tubules, as glucose and uric acid utilize shared transporters such as GLUT9 and URAT1 (38). Moreover, insulin resistance (IR) directly promotes renal sodium and urate reabsorption, while also stimulating hepatic purine catabolism and urate production by activating enzymes such as xanthine oxidase (39). Chronic inflammation and oxidative stress under diabetic conditions further facilitate urate crystal deposition and impair renal excretion (40). Obesity acts as a critical mediator in this process: Visceral adipose tissue secretes free fatty acids and pro-inflammatory cytokines (such as IL-6 and TNF-α), which not only exacerbate hepatic IR but also impair renal urate excretion (41, 42). Additionally, obesity exacerbates insulin resistance, which in turn triggers hyperinsulinemia and consequently diminishes the clearance of uric acid (43).

This analysis contributes substantially to the field by extending the existing evidence base from hyperuricemia to clinically diagnosed gout in patients with diabetes. By focusing on the CVAI, an ethnicity-specific, validated measure of visceral adiposity in Chinese adults, our findings demonstrate that the presence of visceral fat, as quantified by CVAI, is independently linked to gout among individuals with T2DM. Several methodological strengths distinguish this work. These include the large, well-characterized cohort drawn from two specialized metabolic centers; evaluation of CVAI as both continuous and categorical variables; and comprehensive adjustments for key metabolic and renal covariates such as HbA1c, β-cell function (FCp), and serum uric acid. Moreover, the maintained strength of the link in various subgroups enhances the credibility and generalizability of the findings. From a clinical perspective, the results have several important implications. First, CVAI provides a simple, cost-effective, and easily obtainable metric for assessing visceral adiposity, which could support early risk stratification for gout in patients with T2DM, particularly in settings where imaging-based VAT quantification is not feasible. Second, these findings reinforce the concept that visceral adiposity represents a modifiable risk factor for gout. Interventions aimed at reducing visceral fat, through structured lifestyle modification or pharmacologic therapy, may help lower gout risk in this vulnerable population. Finally, identifying patients with elevated CVAI enables clinicians to intensify serum urate monitoring and initiate preventive measures before the onset of clinical gout.

Future research should further evaluate whether CVAI-guided risk management translates into improved clinical outcomes. Prospective cohort studies and randomized controlled trials targeting visceral fat reduction would be particularly valuable in establishing causal pathways and optimizing prevention strategies for gout in diabetic populations.

Limitations to this study must also be acknowledged. First, our participants were recruited from urban hospitals in Eastern China, potentially limiting generalizability to other ethnicities and regions. Second, despite adjustments for numerous demographic and metabolic factors, residual confounders cannot be excluded, particularly regarding physical activity (44) and genetic susceptibility (45). Third, the cross-sectional nature of the investigation precludes causal extrapolation, and longitudinal studies are required to establish the utility of elevated CVAI in predicting incident gout in T2DM patients.

## 5 Conclusion

To conclude, this investigation showed that the CVAI is independently and positively linked with gout prevalence in Chinese adults with T2DM. These results demonstrate the contribution of visceral adiposity in the complex metabolic milieu of diabetes and position CVAI as a practical, readily applicable tool for identifying individuals at elevated risk of gout. Integrating CVAI into routine clinical risk assessment may enable earlier detection and more personalized preventive strategies, particularly those targeting visceral fat reduction. Future longitudinal and interventional analyses are required to verify the predictive utility of CVAI for incident gout and to determine whether interventions to reduce visceral adiposity can effectively lower gout risk in this vulnerable population.

## Data Availability

The original contributions presented in the study are included in the article/Supplementary material, further inquiries can be directed to the corresponding author.

## Supplementary material

Supplementary Figure 1 Relationship between CVAI and gout prevalence in male **(A)** or female **(B)**. Adjusted for age, sex, education level, smoking, alcohol consumption, SBP, FCp, HbA1c, UA, and eGFR. Only the central 99% of the data distribution is shown.
