# Supplementary material for: Relationship between the Chinese visceral adiposity index and gout in individuals with type 2 diabetes mellitus: a cross-sectional population-based study
Source: Front Nutr. 2025 Nov 21;12:1697822. doi: 10.3389/fnut.2025.1697822 (PMC12678096; doi:10.3389/fnut.2025.1697822)
Supplement: Supplementary file 1 [file Table_1.docx]

**Supplementary Table 1 Incremental predictive value of CVAI and related indices for gout in T2DM participants**

|  | **C-statistic**  **(95%CI)** | **P value** | **Continunous NRI**  **(95%CI)** | **P value** | **IDI (95%CI)** | **P value** |
| --- | --- | --- | --- | --- | --- | --- |
| Basic model | 0.788(0.773, 0.804) | Ref | Ref | Ref | Ref | Ref |
| Basic model + CVAI | 0.796(0.781, 0.811) | < 0.001 | 0.2476[0.1764-0.3187] | < 0.001 | 0.004[0.0018-0.0062] | 0.00037 |
| Basic model + VAI | 0.789(0.773, 0.805) | 0.099 | 0.1347[0.0671-0.2023] | 9e-05 | 8e-04[-4e-04-0.0019] | 0.18442 |
| Basic model + BMI | 0.794(0.778, 0.809) | 0.004 | 0.2168[0.1455-0.2882] | < 0.001 | 0.0038[0.0016-0.006] | 0.00064 |
| Basic model + WC | 0.794(0.779, 0.809) | 0.001 | 0.2056[0.1342-0.2769] | < 0.001 | 0.0028[0.001-0.0047] | 0.00312 |

Basic model adjustment included: age, gender, education level, smoking, alcohol consumption, SBP, FCp, HbA1c, UA, and eGFR. Abbreviations: NRI, net reclassification improvement; Ref, reference; IDI, integrated discrimination improvement; CI, confidence interval; Ref, reference.
