# Supplementary figures and images for: Relationship between the Chinese visceral adiposity index and gout in individuals with type 2 diabetes mellitus: a cross-sectional population-based study
Source: Front Nutr. 2025 Nov 21;12:1697822. doi: 10.3389/fnut.2025.1697822 (PMC12678096; doi:10.3389/fnut.2025.1697822)

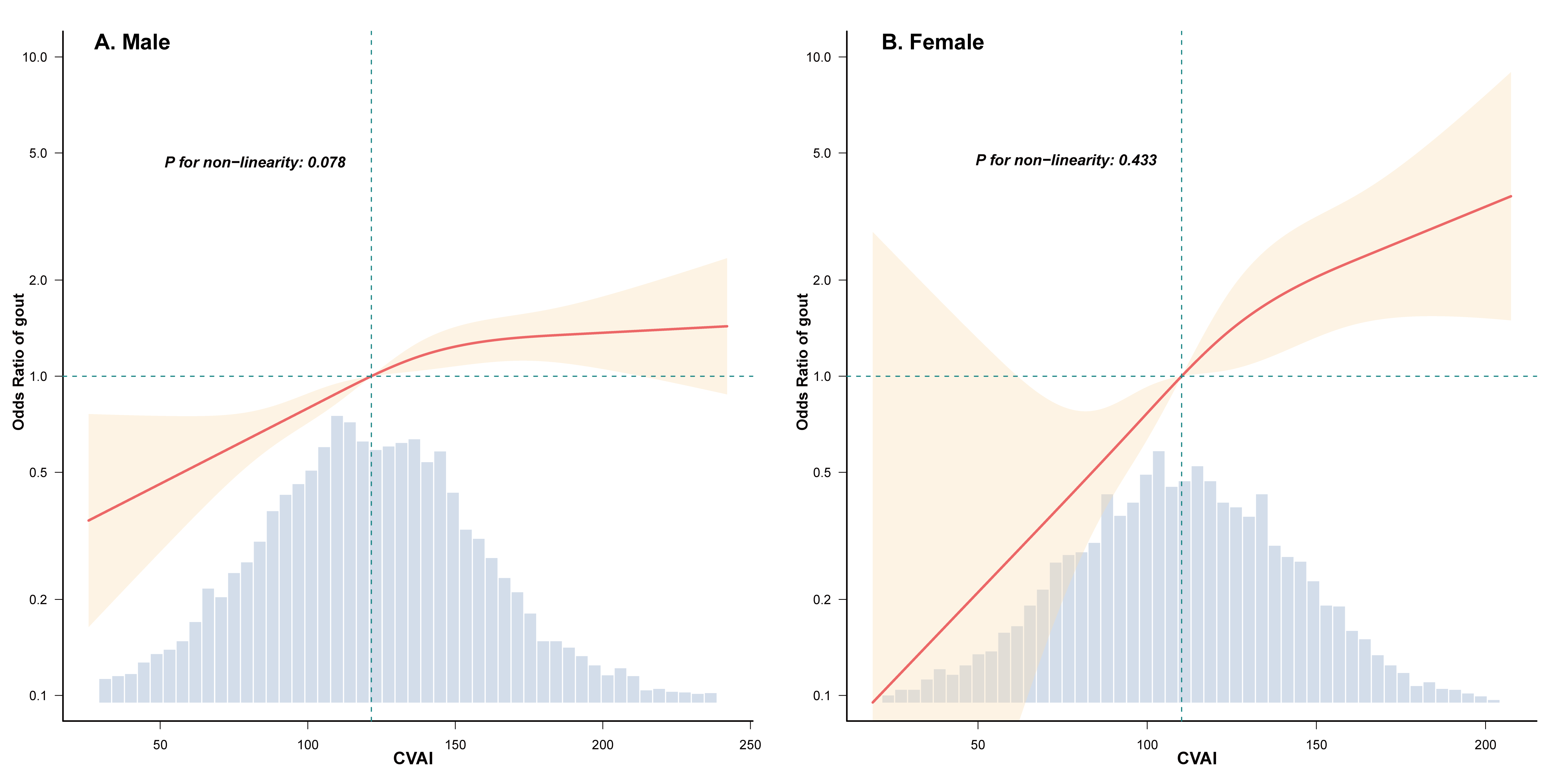

Supplement: Supplementary Figure 1 — Relationship between CVAI and gout prevalence in male (A) or female (B). Adjusted for age, sex, education level, smoking, alcohol consumption, SBP, FCp, HbA1c, UA, and eGFR. Only the central 99% of the data distribution is shown. [file Image_1.tif]
